# Herpes simplex virus 1 induces egress channels through marginalized host chromatin

**DOI:** 10.1038/srep28844

**Published:** 2016-06-28

**Authors:** Markko Myllys, Visa Ruokolainen, Vesa Aho, Elizabeth A. Smith, Satu Hakanen, Piritta Peri, Anna Salvetti, Jussi Timonen, Veijo Hukkanen, Carolyn A. Larabell, Maija Vihinen-Ranta

**Affiliations:** 1 University of Jyvaskyla, Department of Physics and Nanoscience Center, Jyvaskyla, FI-40500, Finland; 2 University of Jyvaskyla, Department of Biological and Environmental Science and Nanoscience Center, Jyvaskyla, FI-40500, Finland; 3 Department of Anatomy, University of California San Francisco, San Francisco, CA, 94158, USA; 4 Physical Biosciences Division, Lawrence Berkeley National Laboratory, Berkeley, CA, 94720, USA; 5 University of Turku, Turku, Finland; 6 International Center for Research in Infectiology (CIRI), INSERM U1111, CNRS UMR5308, Lyon, F-69007, France; 7 Ecole Normale Supérieure de Lyon, Lyon F-69007, France; 8 Université de Lyon, UCB-Lyon1, Lyon, F-69007, France; 9 LabEx Ecofect, Université de Lyon, Lyon, F-69007, France; 10 ITMO University, Kronverkskii ave. 49, 197101, Saint Petersburg, Russia

## Abstract

Lytic infection with herpes simplex virus type 1 (HSV-1) induces profound modification of the cell nucleus including formation of a viral replication compartment and chromatin marginalization into the nuclear periphery. We used three-dimensional soft X-ray tomography, combined with cryogenic fluorescence, confocal and electron microscopy, to analyse the transformation of peripheral chromatin during HSV-1 infection. Our data showed an increased presence of low-density gaps in the marginalized chromatin at late infection. Advanced data analysis indicated the formation of virus-nucleocapsid-sized (or wider) channels extending through the compacted chromatin of the host. Importantly, confocal and electron microscopy analysis showed that these gaps frequently contained viral nucleocapsids. These results demonstrated that HSV-1 infection induces the formation of channels penetrating the compacted layer of cellular chromatin and allowing for the passage of progeny viruses to the nuclear envelope, their site of nuclear egress.

Herpes viruses target the cell nucleus because of their need for the cellular DNA reproduction machinery. The nuclear entry of viral DNA is followed by the nuclear accumulation of viral proteins and replication of viral DNA, leading to the formation of viral replication compartments (VRCs). Electron microscopy and confocal microscopy studies have illustrated how profoundly these viruses transform the nuclear structure so as to optimise their multiplication^1^,^2^,^3^,^4^,^5^.

During cellular entry the nucleocapsid of herpes simplex virus type-1 (HSV-1) undergoes partial disassembly at the nuclear pore complex (NPC), followed by viral genome delivery via the pore^6^,^7^. When the infection proceeds, the VRCs emerge as distinct nuclear foci, which subsequently undergo fusion and expansion into a large, globular VRC^8^,^9^,^10^,^11^. The infected cell expresses and multiples only a limited number of input HSV-1 genomes (fewer than eight at a multiplicity of infection of 100)^12^. Each small VRC, initially located at the nuclear periphery, originates from a single viral genome^12^,^13^,^14^,^15^. These small VRCs undergo actin-mediated directed movement^8^, resulting in their fusion and eventual enlargement into the entire nucleus. The expansion of the VRCs is accompanied by an increase in the nuclear volume and relocation of the host chromatin into the nuclear periphery^16^,^5^. It is in these VRCs that viral nucleocapsid assembly occurs. Soon after assembly, each nucleocapsid penetrates the host chromatin layer and nuclear lamina to the inner nuclear envelope, the site of its egress^2^,^17^,^18^.

Despite their many achievements with biological applications, light- and electron-based imaging techniques suffer from fundamental limitations in 3D imaging of the subcellular architecture of the entire cell. Transmission electron microscopy (TEM) and TEM tomography are limited by fixation-induced distortions of cellular features, damage caused by the electron beam and the limited range of angular sampling. Fluorescence microscopy can determine the positions of specific molecules, but only of those selected based on existing information, including the sizes of the labels and the density of the labelling. Furthermore, immunofluorescence imaging is prone to artefacts by fixation or permeabilization^19^,^20^.

We employed a strategy integrating 3D soft X-ray tomography (SXT) imaging with cryogenic fluorescence microscopy (CFM), confocal and electron microscopy, and advanced data analysis in order to study in more detail the HSV-1-induced changes in nuclear architecture, molecular organization of the host chromatin and, in particular, the formation of channels across the chromatin layer. The short wavelength of soft X-rays enabled high-resolution 3D imaging of the nuclear architecture. Moreover, since X-rays can penetrate 15 μm-thick biological material, SXT allows for quantitative assessment of the entire cell. For SXT image acquisition, the cells were placed in (cylindrical) thin-walled capillaries of diameter up to 15 μm. B cells were used because of their small size and HSV-1 susceptibility^21^,^22^.

In fact, SXT permits cell imaging in a near-native state i.e. intact, unsliced, unstained, and fully hydrated. X-ray absorption depends on the concentration of organic material in each voxel. Therefore, SXT not only detects multiple cellular structures but can also provide quantitative assessment of their composition and structure. Contrast in soft X-ray microscopy is generated by differential attenuation of X-rays by the biomolecules in the specimen and is not muted by weakly absorbing water. Attenuation of X-rays by the specimen follows the Beer-Lambert law^23^ and is therefore both a linear and a quantitative measure of the thickness and the chemical species present at each point in the cell^24^,^25^,^26^. To gain insight into the spatial localization of the host chromatin and of the viral and cellular proteins and nucleocapsids, we complemented 3D SXT with confocal and electron microscopy imaging techniques.

## Results

### Infectivity of B cells

To follow the progress of infection, we analysed the expression of viral immediate-early, early and late genes by cytometry and real-time RT-PCR (qPCR) and of the virus yields by plaque assay^27^,^28^,^29^,^30^. We detected the presence of viral proteins, as well as the expression of viral lytic genes of all three phases of the replication cycle and substantial production of the progeny virus from the B cells. The highest yield of virus was obtained at 24 h p.i. (Supplementary Figs S1 and S2; see also Supplementary Text S1 for additional information). This suggests that not only could HSV-1 enter and infect B cells but also that its replication cycle was completed. Based on these findings, we decided to use a multiplicity of infection (MOI) of 5 and the time point 24 h p.i. in our subsequent experiments.

### Virus-induced reorganization of the host chromatin

To further analyse the nuclear architecture, we used SXT on hydrated cells in their near-native state. The image contrast of SXT is based on the absorption of X-rays by mainly carbon and nitrogen. This allows measurement of the linear absorption coefficients (LAC) of cellular structures reflecting their concentration of cellular biomolecules^31^,^32^. Owing to its high density of biomolecules, the heterochromatin region of the nucleus has a er LAC than the less densely packed nucleoplasm, as is evident from computer-generated SXT ortho-slices through nuclei (Supplementary Fig. S3). The use of an HSV-1 strain expressing EYFP-ICP4 allowed the detection of infected cells with enlarged VRC by CFM used in SXT studies. When SXT ortho-slices were aligned with CFM images of the same cell^33^,^34^, EYFP-ICP4 was found to be localized in distinct nuclear foci or in a few enlarged foci in the heterochromatin-depleted nuclear regions (Supplementary Fig. S3).

### Virus-induced gaps in the cellular chromatin

**Figure 1:**
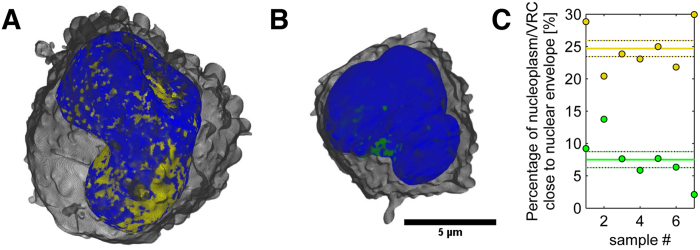
Structure of the marginalized host chromatin. Volume-rendered 3D views of nuclei, showing (**A**) the high-LAC value heterochromatin (blue) and low-LAC value nucleoplasm with VRC (yellow) of an infected cell, and (**B**) the heterochromatin (blue) and nucleoplasm without VRC (green) of a non-infected cell. See also Supplementary Video S1. (**C**) Percentage of nucleoplasm close to the nuclear envelope in infected (with VRC, yellow) and non-infected (green) cells. Solid lines represent the mean and dotted lines the mean ± SEM (n = 7).

The distinct LAC values of SXT were used to automatically segment the nuclear structures for 3D visualization of the spatial information in HSV-1 infected cells. Surface-rendered 3D tomographic reconstructions (Fig. 1A; Supplementary Video S1) of the nuclear periphery of the infected cells revealed low-density gaps in the compact layer of marginalized host chromatin. Statistical analysis showed that 24.7 ± 1.2% (n = 7) of the surface area (10 closest voxels with a linear voxel size of 320 nm) of the chromatin had a low-LAC value (0.222–0.322 μm^−1^), suggesting the presence of low-density regions in the compact chromatin (Fig. 1C). In the non-infected cells the relative area of the region with a low LAC value was 7.5 ± 1.2% (n = 7) (Fig. 1B,C).

**Figure 2:**
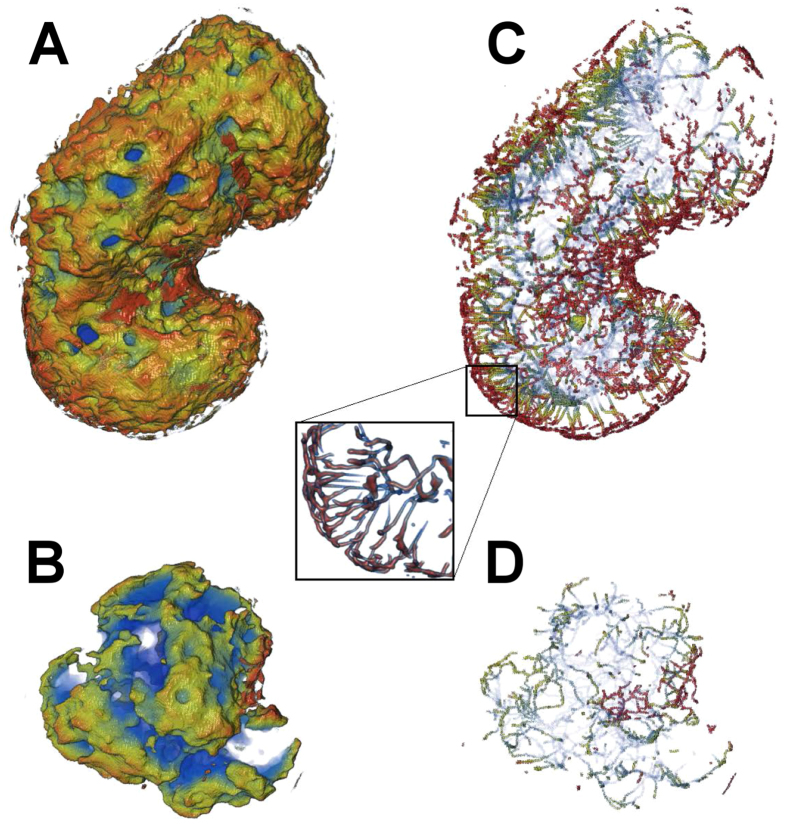
Distribution of low-LAC channels in close proximity to the nuclear envelope in infected and non-infected cells. Low-LAC regions of the nucleoplasm with (**A**) and without (**B**) VRC adjacent to the nuclear envelope. A computationally reduced skeletonized structure of the low-LAC regions in the 0.5 μm-thick layer of host chromatin close to the nuclear envelope, which shows channels across the peripheral heterochromatin in infected (**C**) as well as non-infected (**D**) cells. Pseudo-colour indicates (increasing from red to blue) the distance from the nuclear envelope. See also Supplementary Video S2.

**Figure 3:**
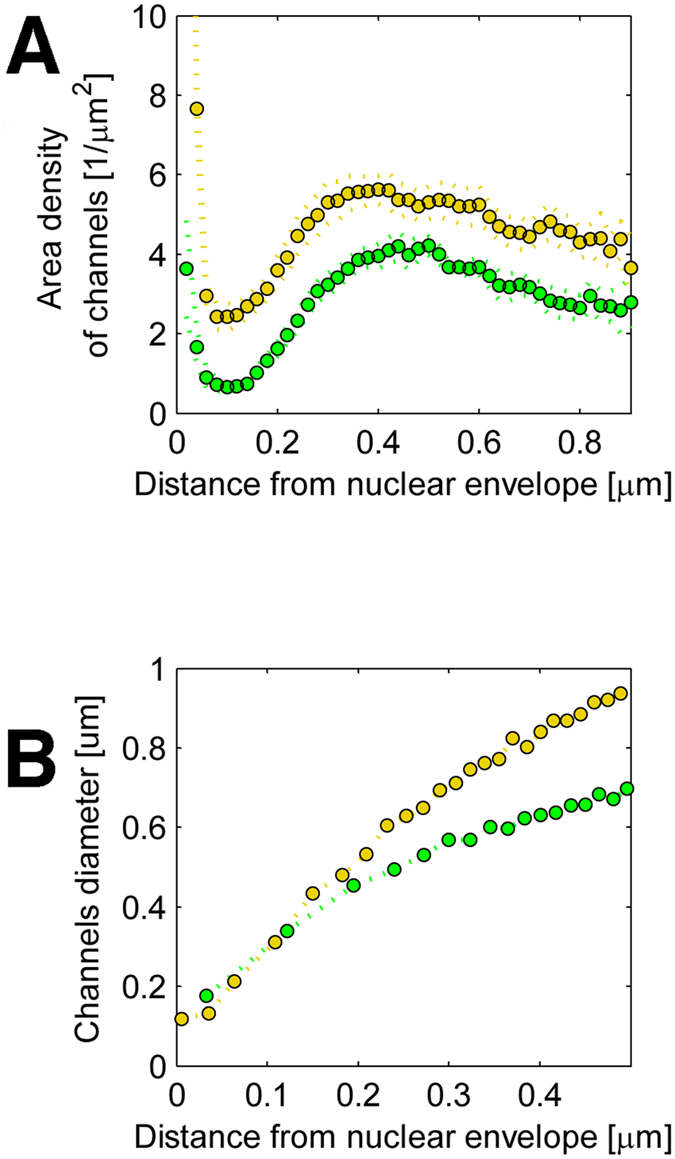
Density and diameter of low-LAC channels next to nuclear envelope. (**A**) Analysis of SXT images, showing the area density of channels across the heterochromatin as a function of distance from the nuclear envelope for infected (yellow) and non-infected (green) cells. (**B**) Local thickness of the channels as a function of distance from the nuclear envelope. Dotted lines represent the means of the distributions (n = 7).

In order to further study the low-LAC regions, we analysed their ‘skeletonized’ versions. The skeletonized structure revealed that the low-LAC regions formed channels in the 0.5 μm-thick layer of host chromatin close to the nuclear envelope, some of which penetrated the nuclear periphery across the layer of peripheral (compacted) chromatin in both infected and non-infected cells (Fig. 2A–D: see also Supplementary Video S2). However, in the infected cells the total number of low-LAC breaks, 900 ± 300 (n = 7), was significantly higher (p < 0.01) than in the non-infected cells (170 ± 40, n = 7). Note that the average volume of the nucleus (260 ± 20 μm^3^), was significantly (p < 0.01) increased in the infected cells compared to the non-infected cells (170 ± 14 μm^3^). Additionally, analysis of the density of these channels across the layer of marginalized heterochromatin as a function of distance from the nuclear envelope (SXT images) revealed that, in a 0.1 μm band close to the nuclear envelope, the area density of these channels was significantly (p < 0.01) higher in the infected (2.4 ± 0.4 μm^−2^) than in the non-infected (0.65 ± 0.11 μm^−2^) cells (Fig. 3A). Furthermore, analysis of their local thickness as a function of distance from the nuclear envelope indicated that their diameter increased towards the nuclear centre, and that they finally merged with the nucleoplasm of the infected (with a VRC) and non-infected cells. The smallest diameter of these channels at the nuclear periphery, at 0.1 μm from the nuclear envelope in both cases, was at least 200 nm (Fig. 3B). Their size was thus sufficient to allow the passage of at least one viral nucleocapsid (diameter 125 nm) at a time.

**Figure 4:**
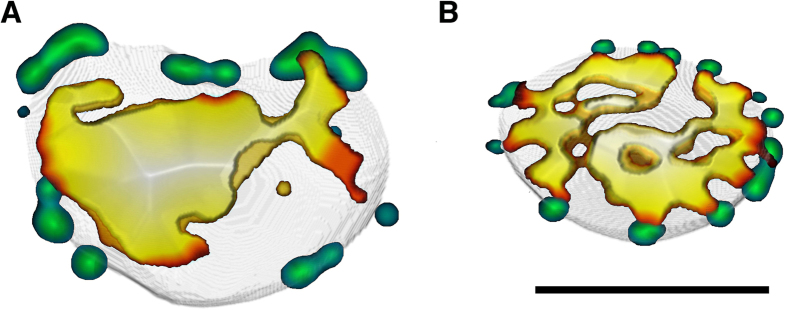
Spatial interaction between low-density gaps and nuclear pore complexes. Views of nuclear middle section, showing 3D reconstructions of confocal images of (**A**) infected and (**B**) non-infected cells. Images show the distribution of low-DAPI areas in comparison to Nup153-labeled NPCs (green). Low-DAPI areas denote DAPI intensity <23% of maximum. In the colour map, the low-DAPI area ranges from blue to white. Blue indicates a minimal distance whereas white indicates a maximal distance from the nuclear envelope. Scale bars, 3 μm.

It is known that heterochromatin-free gaps are associated with NPCs and involved in nucleo-cytoplasmic transport of non-infected cells^35^. This prompted us to study whether the virus-induced channels are connected with NPCs. The number and distribution of low-DAPI gaps across the heterochromatin were compared with those of NPCs. Immunolabelling of cells with the Nup153 antibody revealed that NPCs frequently formed clusters in both infected and non-infected cells. Some of these clusters were significantly enlarged in the infected cells (Fig. 4A; see also Supplementary Fig. S4). The numbers of NPCs or NPC clusters in the infected cells (105 ± 6, n = 19) were reduced compared to those in non-infected (145 ± 9, n = 20) cells (Fig. 4A,B; see also Supplementary Fig. S4). Accordingly, in the infected cells the total number of low-LAC breaks was higher than the number of NPCs, whereas in the non-infected cells the number of breaks in the nuclear periphery was close to that of NPCs. Moreover, our studies showed that, in the infected cells, the low-DAPI regions were almost always located independently of NPCs, whereas in the non-infected cells, the low-DAPI regions were located adjacent to the NPCs (Fig. 4A,B).

In summary, these results demonstrated that HSV-1 infection increases the number of virus-capsid-sized and NPC-independent gaps, which may facilitate the viral transport across the compacted layer of chromatin.

### Nucleocapsids in low-density chromatin gaps

**Figure 5:**
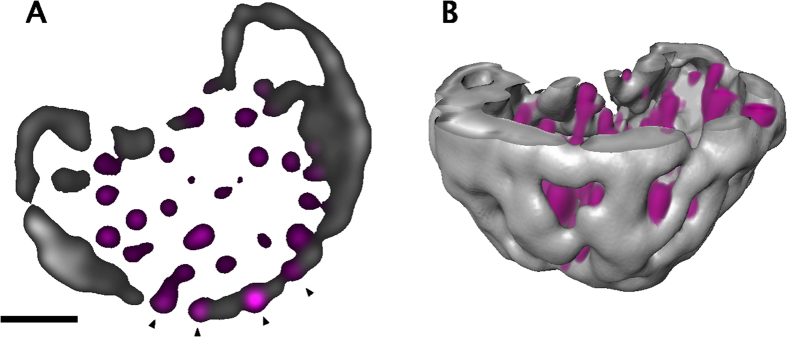
Distribution of nucleocapsid proteins in low-density chromatin regions of nuclear periphery. (**A**) Confocal microscopy images of an infected cell with marginalized chromatin stained with antibodies for the capsid protein VP5 (magenta) and DAPI (grey). White arrowheads indicate the presence of VP5 in the low-DAPI regions (black) in close proximity to the nuclear envelope. Scale bar is 3 μm. (**B**) 3D reconstruction of confocal image, which shows VP5 (magenta) and chromatin (grey). See also Supplementary Video S3.

Confocal microscopy was performed to detect the presence of nucleocapsid proteins in the low-density chromatin regions of the nuclear periphery. Viral capsid protein VP5 was frequently seen in low-DAPI regions in close proximity to the nuclear envelope (Fig. 5; see also Supplementary Video S3).

**Figure 6:**
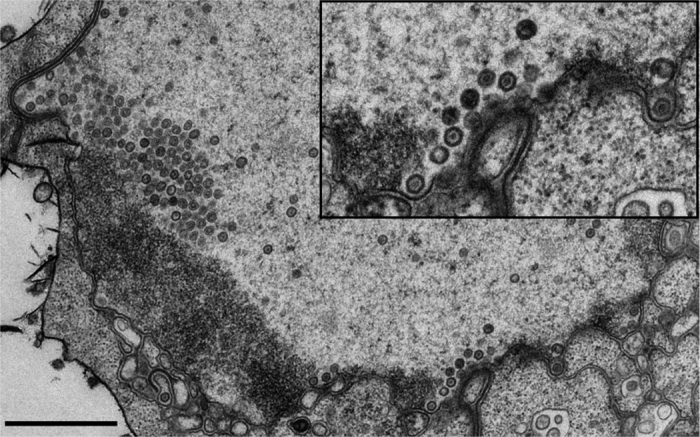
Nucleocapsids in low-density regions of chromatin. Transmission electron microscopy images of infected cell nuclei, which show B cells at 24 h p.i. The inset shows enlarged view of the boxed areas containing the viral nucleocapsids adjacent to the nuclear envelope. Scale bar, 1 μm.

Next, we used transmission electron microscopy to examine the distribution of nucleocapsids with respect to the peripheral marginalized chromatin. The images showed that nucleocapsids were very often localized in the chromatin region next to the nuclear envelope. Consistently with the confocal data, these nucleocapsids were located in the low-density chromatin breaks and narrow virus-nucleocapsid-sized channels (Fig. 6A). The NPCs are mostly invisible in the infected cells, presumably because of virus induced structural changes of the NE. The distribution of chromatin and NPCs in the non-infected cells is shown in the Supplementary Fig. S5. This is in line with recent studies showing that herpesvirus infection increases the porosity of the nucleus, leading to an enhancement of nucleocapsid motility^36^. Altogether, our findings showed that HSV-1 infection induces breakages penetrating the cellular chromatin barrier to permit nucleocapsid access to the nuclear envelope.

## Discussion

Formation of a VRC as a result of HSV-1 lytic infection is followed by structural changes in the host chromatin^5^,^37^,^38^,^39^. Profound reorganization of the nuclear chromatin by HSV-1 has been known to include chromatin marginalization to the nuclear periphery^16^,^5^. A compact layer of host heterochromatin constitutes an accessibility barrier for the translocation of viral nucleocapsids toward the inner nuclear envelope across which they exit the nucleus. A previous indication of HSV-1 infection’s ability to disrupt marginalized chromatin, so as to allow access through it, has come from immunofluorescence studies showing fragmented distribution of histone H1, suggesting the presence of routes through chromatin^5^. By combining SXT, CFM and confocal imaging techniques with advanced image analysis, we achieved a new insight into the 3D structure and distribution of such breakages.

We observed that the low-density regions in the host chromatin formed gaps through it. In the absence of infection these openings were typically located near NPCs, in agreement with earlier results on the density distribution of the host chromatin^35^. In the infected cells, the number of these low-density breakages was significantly increased, and they were most often located independent of NPCs. This independence was in agreement with the NPC-independent nuclear egress of the virus^2^,^40^. The SXT analysis revealed channels wide enough to allow the passage of a 125-nm-wide HSV-1 nucleocapsid. However, the narrowest channels, with a diameter of 200 nm, allowed the passage of only one or a few nucleocapsids at a time. This leads to the presence of nucleocapsids in the discontinuous-density regions of the peripheral chromatin close to the nuclear envelope, which agrees well with the egress mechanism demonstrated previously^2^,^5^. The exact nature of the molecular mechanisms involved in the intra-nuclear motility of the capsids is controversial. It has been suggested that nucleocapsids are transported in the nucleoplasm via an active process mediated by an intra-nuclear actin and myosin motor protein^3^,^41^,^42^,^43^,^44^,^45^. However, a very recent study showed that their motility is based on passive diffusion^36^,^46^.

In summary, we were able to create 3D reconstructions of intact and fully hydrated HSV-1 infected cell nuclei by using SXT. We also used other techniques to complement the structural information thus gained. Our study reveals the induction of channels allowing for the nuclear egress of the progeny viruses across the host chromatin. Moreover, this work used a new combination of methods in the study of virus-cell interactions.

## Materials and Method

### Cells and viruses

A continuously growing pre-B cell line was produced with the Abelson murine virus^47^. The female mouse B cells were a gift from Barbara Panning (UCSF School of Medicine, Biochemistry and Biophysics, San Francisco, CA). The Epstein-Barr virus-transformed human B lymphocytes (GM12878) were purchased from the NGIMS Human Genetics Cell Repository, Coriell Institute of Medical Research (NJ). The mouse B and human B cells were maintained as suspension cultures at 37 °C in RPMI-1640 medium (GIBCO, Invitrogen, Inc.), supplemented with 10% or 15% of fetal bovine serum (FBS) (GIBCO, Invitrogen, Inc.), L-glutamine, penicillin, streptomycin and 5% CO_2_. The medium was refreshed every two to three days to maintain a cell density of 0.8–2 × 10^6^ cells/ml. The HSV-1 strains were wt (17+) and 17+ expressing EYFP-ICP4 (vEYFP-ICP4), a generous gift from R. Everett (MRC Virology Unit, Glasgow, Scotland, UK)^1^. The viruses had been isolated as described in^1^. For infection, cells were inoculated with HSV-1 or HSV-1 EYFP-ICP4 (MOI 5–10) and kept at 37 °C until the SXT analysis, live-cell microscopy or fixation.

### Immunolabelling studies

The cells were infected with HSV-1 or vEYFP-ICP4 HSV-1 at MOI 5. At 24 h p.i., cells were collected by centrifugation (400 RCF for 5 min), spread and air-dried on Zeiss high-performance cover slips (D = 0.17 mm, size 18 × 18 mm^2^). Cells were fixed with 4% paraformaldehyde (PFA; 20 min at room temperature [RT]). Viral capsid proteins were detected with a capsid protein VP5-specific monoclonal antibody (MAb; Santa Cruz Biotechnology, Inc., TX) and NPCs with Nup153 MAb (Abcam, Cambridge, MA), followed by a goat anti-mouse Alexa 594 secondary antibody (Ab; Molecular Probes, Life Technologies, NY). DNA was stained during embedding with DAPI (4′-6-diamidino-2-phenylindole; Molecular Probes), containing ProLong antifade compound. Imaging was done using a Nikon A1R laser-scanning confocal microscope (Nikon Instruments, Inc., Amsterdam, Netherlands.) with the CFI Plan Apo VC 60XH (NA 1.4, WD 0.13, oil immersion objective). DAPI was excited with a 405-nm diode laser and its fluorescence was detected with a 450/50-nm band-pass filter. EYFP was excited with a 514-nm argon laser and its fluorescence was collected with a 540/30-nm band-pass filter. Alexa 594 was excited with a 561-nm sapphire laser and the fluorescence was collected with a 595/50-nm band-pass filter. For 3D image stacks, 512 × 512 pixels were collected from an appropriate depth depending on the sample. Pixel resolution was adjusted to 50 nm/pixel in x and y dimensions, and to 150 nm in the z dimension. AutoGain was used in multipoint imaging. Iterative deconvolution was performed with a signal-to-noise ratio set at 7, a quality threshold set at 0.0l. Deconvolution was performed with Huygens Essential software (SVI, Hilversum, Netherlands). Image analysis was done with ImageJ^48^. The number of NPCs was calculated from the maximum intensity projection images. The amount and volume of NPC areas were calculated by a 3D object counter from manually thresholded data. In the fixed-cell images the volume analysis was done by first de-noising the images with a Gaussian blur, sigma = 2, and then making a binary image with the Otsu threshold. Holes were filled and the amount and volume of objects were determined using the 3D-Objects-Counter plugin^49^.

### Specimen preparation for soft X-ray tomography

Cells were seeded at a concentration of 10^6^ cells/ml into a standard plastic culture flask (Corning, Corning, NY), and infected with vEYFP-ICP4 at a MOI of 5 for 24 h at 37 °C. Infected cells were pelleted by centrifugation (400 RCF for 5 min), re-suspended in the growth medium, filtered, loaded into thin-walled glass capillaries and then vitrified by quickly plunging them into liquid-nitrogen-cooled (~90 °K) liquid propane, using a custom device^34^.

### Cryogenic confocal fluorescence microscopy

Vitrified cells expressing EYFP-ICP4 were identified and imaged in specimen capillaries, using a custom-made cryogenic confocal fluorescence microscope. The cryogenic light microscope, made in-house, features a custom-made specimen holder, a modified immersion objective lens and a ~10 l reservoir of liquid nitrogen. Other components were commercial, including a Yokogawa spinning-disk confocal head (CSU-X1, Yokogawa, Tokyo, Japan) and an integrated system of acousto-optical tuneable filtered (AOTF) lasers (Andor Laser Combiner, Model LC-501A; Andor Technologies, Belfast, UK). For more detailed description of the instrument, see^34^. EYFP was excited using a 491-nm laser and imaged with an EMCCD (iXon Model Numbers DV887ECS-BV, Andor Technologies) using a standard 580/30-nm band-pass emission filter (Chroma Technology Corp., Bellows Falls, VT).

### Soft X-ray tomography

SXT data were collected with a soft X-ray microscope XM-2 in the National Center for X-ray Tomography (http://ncxt.lbl.gov) at the Advanced Light Source (http://www.als.lbl.gov) of Lawrence Berkeley National Laboratory (Berkeley, CA) using a 50-nm objective zone plate. Cells were kept in a stream of liquid-nitrogen-cooled helium gas^31^,^50^ during data collection, which allowed the collection to be conducted without observable radiation damage. For each data set, 90–180 projection images were collected sequentially around a rotation axis in 1–2° increments, with a total rotation of 180°, using a 300–400 ms exposure time. Projection images were normalized^25^ and manually aligned using the IMOD software, by tracking fiducial markers on adjacent images^51^; tomographic reconstructions were calculated using iterative reconstruction methods^52^,^53^. LAC values were determined as described^54^. The student’s t-test (two-tailed, unequal variance) was used to evaluate statistical significance.

### Transmission electron microscopy (TEM)

Infected cells and non-infected control cells were fixed in 4% paraformaldehyde and 0.25% glutaraldehyde in 50 mM phosphate buffer (pH 6.8) and post-fixed in 1% OsO4 for 1 h on ice. Cells were dehydrated through an ethanol series and then embedded in low-viscosity embedding resin (TAAB Laboratories Equipment Ltd, UK). Thin sections were cut using Ultracut UC6a ultramicrotome (Leica Mikrosysteme GmbH, Germany) and collected on Pioloform-coated, single-slot copper grids. After double staining with 2% aqueous uranyl acetate and lead citrate, the sections were examined using TEM JEOL JEM1400JEOL (JEOL Ltd., Tokyo, Japan), operated at 80 kV. The images were recorded using a bottom-mounted Quemesa CCD camera with 4008 × 2664 pixel resolution.

## Additional Information

**How to cite this article**: Myllys, M. *et al*. Herpes simplex virus 1 induces egress channels through marginalized host chromatin. *Sci. Rep.* **6**, 28844; doi: 10.1038/srep28844 (2016).

## Supplementary Material

Supplementary Information

Supplementary Video 1

Supplementary Video 2

Supplementary Video 3

## References

[b1] Everett R. D., Sourvinos G. & Orr A. Recruitment of herpes simplex virus type 1 transcriptional regulatory protein ICP4 into foci juxtaposed to ND10 in live, infected cells. J. Virol. 77, 3680–3689 (2003). 12610143 10.1128/JVI.77.6.3680-3689.2003 PMC149519

[b2] Johnson D. C. & Baines J. D. Herpesviruses remodel host membranes for virus egress. Nat. Rev. Microbiol. 9, 382–394 (2011). 21494278 10.1038/nrmicro2559

[b3] Reynolds A. E., Liang L. & Baines J. D. Conformational changes in the nuclear lamina induced by herpes simplex virus type 1 require genes U(L)31 and U(L)34. J. Virol. 78, 5564–5575 (2004). 15140953 10.1128/JVI.78.11.5564-5575.2004 PMC415827

[b4] Salvetti A. & Greco A. Viruses and the nucleolus: the fatal attraction. Biochim. Biophys. Acta. 1842, 840–847 (2014). 24378568 10.1016/j.bbadis.2013.12.010 PMC7135015

[b5] Simpson-Holley M., Baines J. D., Roller R. & Knipe D. M. Herpes simplex virus 1 U(L)31 and U(L)34 gene products promote the late maturation of viral replication compartments to the nuclear periphery. J. Virol. 78, 5591–5600 (2004). 15140956 10.1128/JVI.78.11.5591-5600.2004 PMC415826

[b6] Copeland A. M., Newcomb W. W. & Brown J. C. Herpes simplex virus replication: roles of viral proteins and nucleoporins in capsid-nucleus attachment. J. Virol. 83, 1660–1668 (2009). 19073727 10.1128/JVI.01139-08 PMC2643781

[b7] Pasdeloup D., Blondel D., Isidro A. L. & Rixon F. J. Herpesvirus capsid association with the nuclear pore complex and viral DNA release involve the nucleoporin CAN/Nup214 and the capsid protein pUL25. *J. Virol*. 83, 6610–6623 (2009). 19386703 10.1128/JVI.02655-08 PMC2698519

[b8] Chang L. . Herpesviral replication compartments move and coalesce at nuclear speckles to enhance export of viral late mRNA. Proc. Natl. Acad. Sci. USA 108, E136–44 (2011). 21555562 10.1073/pnas.1103411108 PMC3102408

[b9] Kobiler O., Drayman N., Butin-Israeli V. & Oppenheim A. Virus strategies for passing the nuclear envelope barrier. Nucleus 3, 526–539 (2012). 22929056 10.4161/nucl.21979 PMC3515536

[b10] Lukonis C. J. & Weller S. K. Formation of herpes simplex virus type 1 replication compartments by transfection: requirements and localization to nuclear domain 10. *J. Virol*. 71, 2390–2399 (1997). 9032376 10.1128/jvi.71.3.2390-2399.1997 PMC191349

[b11] Simpson-Holley M., Colgrove R. C., Nalepa G., Harper J. W. & Knipe D. M. Identification and functional evaluation of cellular and viral factors involved in the alteration of nuclear architecture during herpes simplex virus 1 infection. J. Virol. 79, 12840–12851 (2005). 16188986 10.1128/JVI.79.20.12840-12851.2005 PMC1235858

[b12] Kobiler O., Lipman Y., Therkelsen K., Daubechies I. & Enquist L. W. Herpesviruses carrying a Brainbow cassette reveal replication and expression of limited numbers of incoming genomes. *Nat. Commun*. 1, 146 (2010). 21266996 10.1038/ncomms1145 PMC3079281

[b13] Kobiler O., Brodersen P., Taylor M. P., Ludmir E. B. & Enquist L. W. Herpesvirus replication compartments originate with single incoming viral genomes. MBio 2, e00278–11 (2011). 22186611 10.1128/mBio.00278-11 PMC3269065

[b14] Silva L., Cliffe A., Chang L. & Knipe D. M. 2008. Role for A-type lamins in herpesviral DNA targeting and heterochromatin modulation. PLos Pathog. 4, e1000071 (2008). 18497856 10.1371/journal.ppat.1000071 PMC2374905

[b15] Silva L. . Roles of the nuclear lamina in stable nuclear association and assembly of a herpesviral transactivator complex on viral immediate-early genes. MBio 3, e00300–11. 22251972 10.1128/mBio.00300-11 PMC3258183

[b16] Monier K., Armas J. C., Etteldorf S., Ghazal P. & Sullivan K. F. 2000. Annexation of the interchromosomal space during viral infection. *Nat. Cell Biol*. 2, 661–665 (2008). 10980708 10.1038/35023615

[b17] Klupp B. G. . Vesicle formation from the nuclear membrane is induced by coexpression of two conserved herpesvirus proteins. Proc. Natl. Acad. Sci. USA 104, 7241–7246 (2007). 17426144 10.1073/pnas.0701757104 PMC1855391

[b18] Park R. & Baines J. D. Herpes simplex virus type 1 infection induces activation and recruitment of protein kinase C to the nuclear membrane and increased phosphorylation of lamin B. J. Virol. 80, 494–504 (2006). 16352573 10.1128/JVI.80.1.494-504.2006 PMC1317514

[b19] Fischer A. H., Jacobson K. A., Rose J. & Zeller R. Fixation and permeabilization of cells and tissues. *CSH Protoc.* pdb top36 (2008). 10.1101/pdb.top36 21356837

[b20] Wilson S. M. & Bacic A. Preparation of plant cells for transmission electron microscopy to optimize immunogold labeling of carbohydrate and protein epitopes. Nat. Protoc. 7, 1716–1727 (2012). 22918389 10.1038/nprot.2012.096

[b21] Spear P. G. & Longnecker R. Herpesvirus entry: an update. J. Virol. 77, 10179–10185 (2003). 12970403 10.1128/JVI.77.19.10179-10185.2003 PMC228481

[b22] Eling D. J., Johnson P. A., Sharma S., Tufaro F. & Kipp T. J. Chronic lymphocytic leukemia B cells are highly sensitive to infection by herpes simplex virus-1 via herpesvirus-entry-mediator A. Gene Ther. 7, 1210–1216 (2000). 10918489 10.1038/sj.gt.3301241

[b23] Hanssen E. . Soft X-ray microscopy analysis of cell volume and hemoglobin content in erythrocytes infected with asexual and sexual stages of Plasmodium falciparum. J. Struct. Biol. 177, 224–232 (2012). 21945653 10.1016/j.jsb.2011.09.003 PMC3349340

[b24] Larabell C. A. & Nugent K. A. Imaging cellular architecture with X-rays. Curr. Opin. Struct. Biol. 20, 623–631 (2012). 10.1016/j.sbi.2010.08.008 PMC3268817 20869868

[b25] Parkinson D. Y., Knoechel C., Yang C., Larabell C. A. & Le Gros M. A. Automatic alignment and reconstruction of images for soft X-ray tomography. J. Struct. Biol. 177, 259–266 (2012). 22155289 10.1016/j.jsb.2011.11.027 PMC3288662

[b26] Parkinson D. Y. . Nanoimaging cells using soft X-ray tomography. Methods Mol. Biol. 950, 457–481 (2013). 23086890 10.1007/978-1-62703-137-0_25

[b27] Nygårdas M. . A herpes simplex virus-derived replicative vector expressing LIF limits experimental demyelinating disease and modulates autoimmunity, PLos One 8, e64200 (2013). 23700462 10.1371/journal.pone.0064200 PMC3659099

[b28] Paavilainen H. . Innate responses to small interfering RNA pools inhibiting herpes simplex virus infection in astrocytoid and epithelial cells. Innate Immun. 21, 349–357 (2015). 24996409 10.1177/1753425914537921

[b29] Romanovskaya A. . Enzymatically produced pools of canonical and Dicer-substrate siRNA molecules display comparable gene silencing and antiviral activities against herpes simplex virus, PLos One 7, e51019 (2012). 23226452 10.1371/journal.pone.0051019 PMC3511422

[b30] Ziegler T. . Typing of herpes simplex virus isolates with monoclonal antibodies and by nucleic acid spot hybridization. J. Virol. Methods 12, 169–177 (1985). 3001118 10.1016/0166-0934(85)90017-5

[b31] McDermott G., Le Gros M. A., Knoechel C. G., Uchida M. & Larabell C. A. Soft X-ray tomography and cryogenic light microscopy: the cool combination in cellular imaging. Trends Cell Biol. 19, 587–595 (2009). 19818625 10.1016/j.tcb.2009.08.005 PMC3276488

[b32] McDermott G. . Visualizing and quantifying cell phenotype using soft X-ray tomography. Bioessays 34, 320–327 (2012). 22290620 10.1002/bies.201100125 PMC3343367

[b33] Smith E. A. . Correlative cryogenic tomography of cells using light and soft x-rays. Ultramicroscopy 143, 33–40 (2014). 24355261 10.1016/j.ultramic.2013.10.013 PMC4013260

[b34] Smith E. A. . The Topological Organization of the Inactive X Chromosome in its Native State. Biophys. J. 106, 434a–435a (2014).

[b35] Raices M. & D’Angelo M. A. Nuclear pore complex composition: a new regulator of tissue-specific and developmental functions. Nat. Rev. Mol. Cell Biol. 13, 687–699 (2012). 23090414 10.1038/nrm3461

[b36] Bosse J. B. . Remodeling nuclear architecture allows efficient transport of herpesvirus capsids by diffusion. Proc Natl Acad Sci. USA 112, E5725–5733 (2015). 26438852 10.1073/pnas.1513876112 PMC4620878

[b37] Knipe D. M. & Cliffe A. Chromatin control of herpes simplex virus lytic and latent infection. Nat. Rev. Microbiol. 6, 211–221 (2008). 18264117 10.1038/nrmicro1794

[b38] Knipe D. M. . M. Snapshots: chromatin control of viral infection. Virology 435, 141–156 (2013). 23217624 10.1016/j.virol.2012.09.023 PMC3531885

[b39] Weller S. K. Herpes simplex virus reorganizes the cellular DNA repair and protein quality control machinery. *PLos Pathog*. 6, e1001105 (2010). 21124825 10.1371/journal.ppat.1001105 PMC2991270

[b40] Nagel C. H. . Nuclear egress and envelopment of herpes simplex virus capsids analyzed with dual-color fluorescence HSV1(17+). J. Virol. 82, 3109–3124 (2008). 18160444 10.1128/JVI.02124-07 PMC2258981

[b41] Tseng Y., Lee J. S., Kole T. P., Jiang I. & Wirtz D. Micro-organization and visco-elasticity of the interphase nucleus revealed by particle nanotracking. *J. Cell Sci*. 117, 2159–2167 (2004). 15090601 10.1242/jcs.01073

[b42] Cano-Monreal G. L., Wylie K. M., Cao F., Tavis J. E. & Morrison L. A. Herpes simplex virus 2 UL13 protein kinase disrupts nuclear lamins. Virology 392, 137–147 (2009). 19640559 10.1016/j.virol.2009.06.051 PMC2769575

[b43] Forest. T., Barnard S. & Baines J. D. Active intranuclear movement of herpesvirus capsids. Nat. Cell Biol. 7, 429–431 (2005). 15803134 10.1038/ncb1243

[b44] Leach N. R. & Roller R. J. Significance of host cell kinases in herpes simplex virus type 1 egress and lamin-associated protein disassembly from the nuclear lamina. Virology 406, 127–137 (2010). 20674954 10.1016/j.virol.2010.07.002 PMC2948959

[b45] Roberts K. L. & Baines J. D. Actin in herpesvirus infection. Viruses 3, 336–346 (2011). 21994736 10.3390/v3040336 PMC3185702

[b46] Bosse J. B. . Nuclear herpesvirus capsid motility is not dependent on F-actin. MBio 7, e01909–14 (2014). 10.1128/mBio.01909-14 PMC4196236 25293761

[b47] D’Andrea E., Saggioro D., Fleissner E. & Chieco-Bianchi L. Abelson murine leukemia virus-induced thymic lymphomas: transformation of a primitive lymphoid precursor. *J. Natl. Cancer Inst*. 79, 189–195 (1987). 3110476

[b48] Abràmoff M. D., Magalhães P. J. & Ram S. J. Image processing with ImageJ. Biophot. internat. 11, 36–43 (2004).

[b49] Bolte S. & Cordelieres F. P. A guided tour into subcellular colocalization analysis in light microscopy. J. Microsc. 224, 213–232 (2006). 17210054 10.1111/j.1365-2818.2006.01706.x

[b50] Le Gros M. A., McDermott G. & Larabell C. A. X-ray tomography of whole cells. Curr. Opin. Struct. Biol. 15, 593–600 (2006). 10.1016/j.sbi.2005.08.008 16153818

[b51] Kremer J. R., Mastronarde D. N. & McIntosh J. R. Computer visualization of three-dimensional image data using IMOD. J. Struct. Biol. 116, 71–76 (1996). 8742726 10.1006/jsbi.1996.0013

[b52] Mastronarde D. N. Fiducial marker and hybrid alignment methods for single-and double-axis tomography. In Electron Tomography , 163–185 (Springer, 2006).

[b53] Stayman J. W. & Fessler J. A. Compensation for nonuniform resolution using penalized-likelihood reconstruction in space-variant imaging systems. IEEE Trans. Med. Imaging 23, 269–284 (2004). 15027520 10.1109/TMI.2003.823063

[b54] Weiss D. . Tomographic imaging of biological specimens with the cryo transmission X-ray microscope. Nuclear Instruments and Methods In Physics Research Section A: Accelerators, Spectrometers, Detectors and Associated Equipment 467, 1308–1311 (2001).

