# Supplementary material for: Herpes simplex virus 1 induces egress channels through marginalized host chromatin
Source: Sci Rep. 2016 Jun 28;6:28844. doi: 10.1038/srep28844 (PMC5378911; doi:10.1038/srep28844)
Supplement: Supplementary Information [file srep28844-s1.pdf]

Herpes simplex virus 1 induces egress channels through marginalized host chromatin

Markko Myllys[1+], Visa Ruokolainen[2+], Vesa Aho[1], Elizabeth A. Smith[3,4], Satu Hakanen[2], Piritta Peri[5], Anna Salvetti[6,7,8,9], Jussi Timonen[1,10], Veijo Hukkanen[5], Carolyn A. Larabell[3,5], and Maija Vihinen-Ranta[2*]

[1]University of Jyvaskyla, Department of Physics and Nanoscience Center, Jyvaskyla, FI-40500, Finland

[2]University of Jyvaskyla, Department of Biological and Environmental Science and Nanoscience Center, Jyvaskyla, FI-40500, Finland

[3]Department of Anatomy, University of California San Francisco, San Francisco, CA, 94158, USA

[4]Physical Biosciences Division, Lawrence Berkeley National Laboratory, Berkeley, CA, 94720, USA

[5]University of Turku, Finland

[6] International Center for Research in Infectiology (CIRI), INSERM U1111, CNRS UMR5308, Lyon, F-69007, France

[7] Ecole Normale Supérieure de Lyon, Lyon F-69007, France

[8] Université de Lyon, UCB-Lyon1, Lyon, F-69007, France

[9] LabEx Ecofect, Université de Lyon, Lyon, F-69007, France

[10] ITMO University, Kronverkskii ave. 49, 197101, Saint Petersburg, Russia

*corresponding. maija.vihinen-ranta@jyu.fi

+these authors contributed equally to this work

SUPPLEMENTAL FIGURES

Figure S1. **Expression of viral genes in B cells**. (A) Expression of HSV-1 proteins in mouse B cells at 24 h.p.i., infected with either at a MOI of 0.5 or 5 of HSV-1 strain (17+). Flow cytometry was used to determine the relative protein-expression levels (mean channel fluorescence) of the HSV immediate early protein ICP0, and the late proteins gC and gD. The percentages of gated positive staining cells are given. (B) Expression of HSV genes in infected mouse B cells. The studied HSV genes were ICP27 (UL54; alpha, immediate-early), ICP8 (UL29; beta, early) and VP16 (alphaTIF or UL48; gamma, late). The expression levels were determined by quantitative RT-PCR from triplicate cultures of cells infected with HSV-1(17+) at a MOI of 0.5 or 5, at time points of 4 and 24 h p.i. The relative expression, normalized to GAPDH expression, is shown in a logarithmic scale for each HSV gene. The average values of triplicates with ±SD are shown.

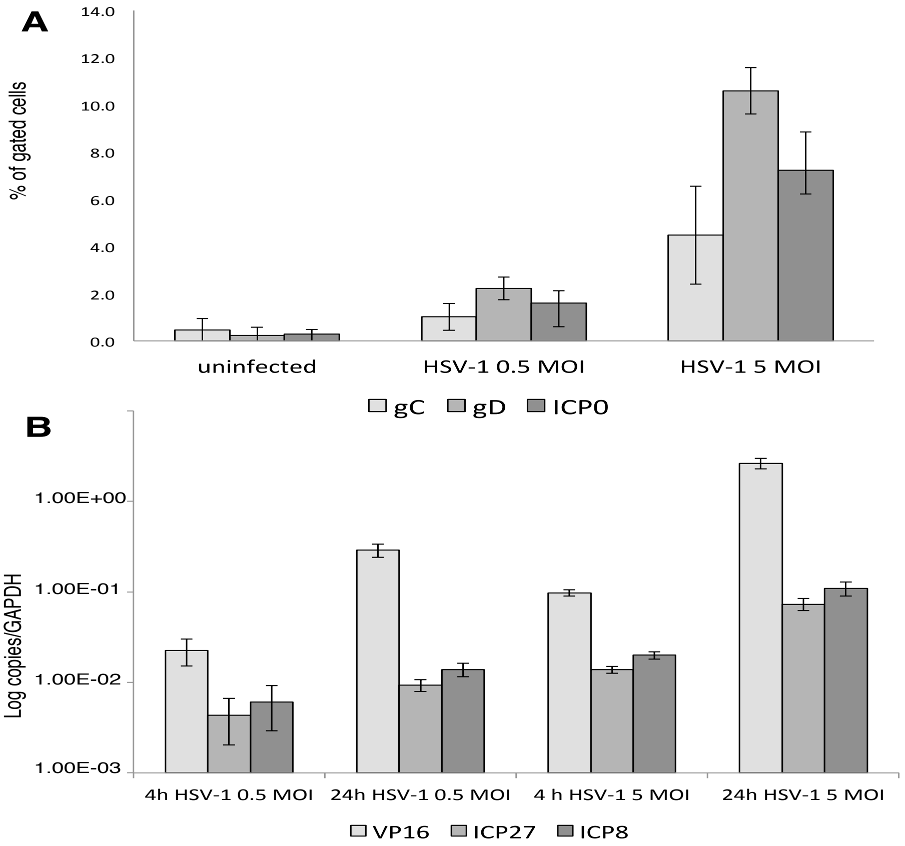

Figure S2. Course of infection in B cells. Shedding of infectious viruses was quantitated from the culture medium of B cells by the plaque titration. The virus yield was assayed from culture supernatants collected at 4, 8, 12, 18, 24, 30, and 48 h p.i. at a MOI of 5. The average values of triplicates with ±SD are shown.

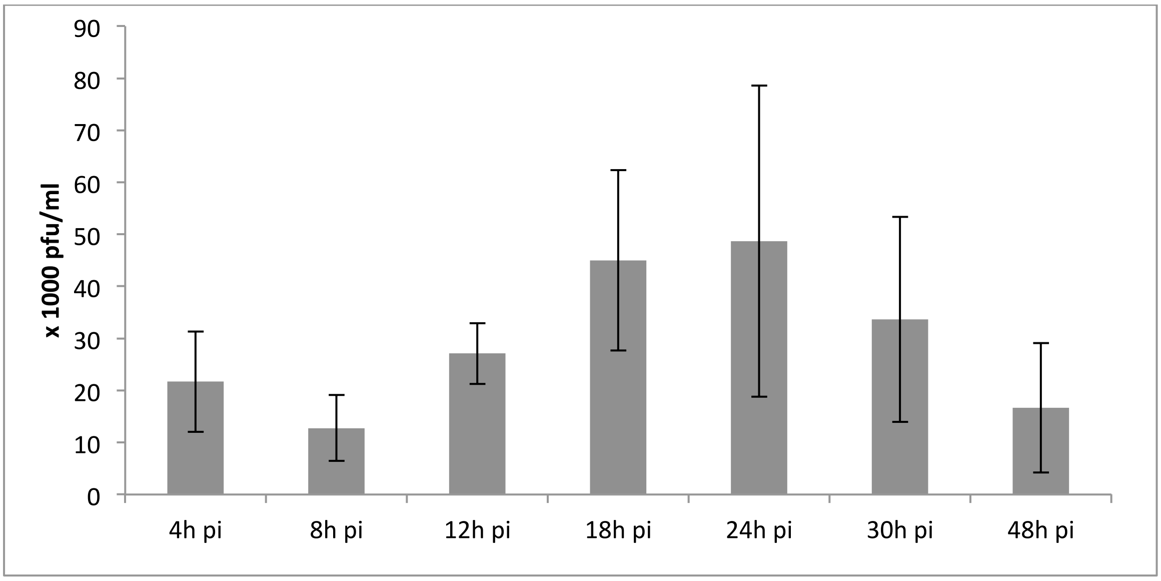

Figure S3. SXT and CFM analysis of nuclear organization of VRC in infected cells. Ortho-slices, or virtual sections, of an X-ray tomographic reconstruction of the infected cell at 24 h p.i. A CFM image of the distribution of viral EYFP-ICP4 (yellow). Scale bar is 3 µm.

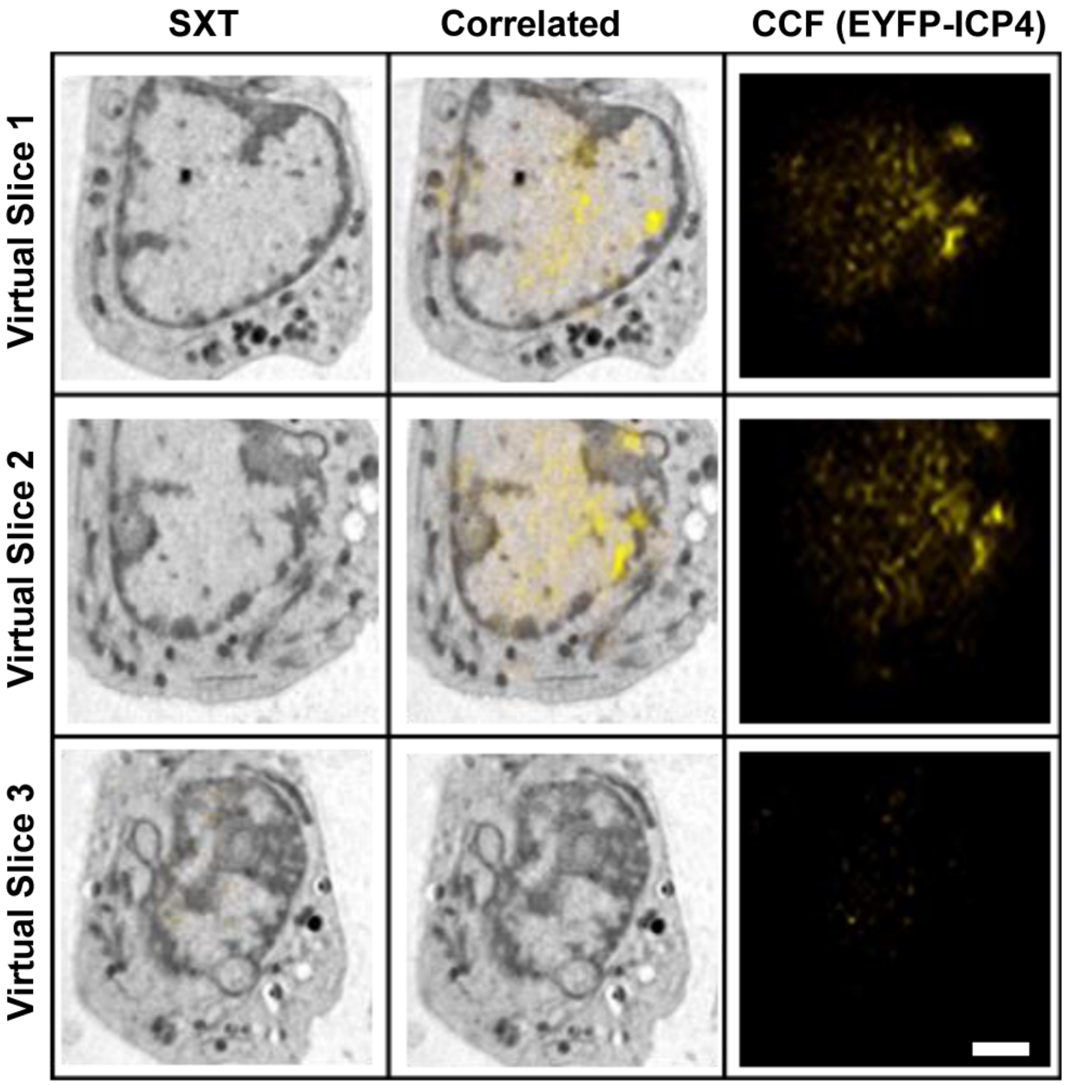

Figure S4. Characterization of NPC regions. Quantitative analysis of confocal data of infected (yellow) and non-infected (green) cells. The number and volume of Nup153-containing NPC areas are shown.

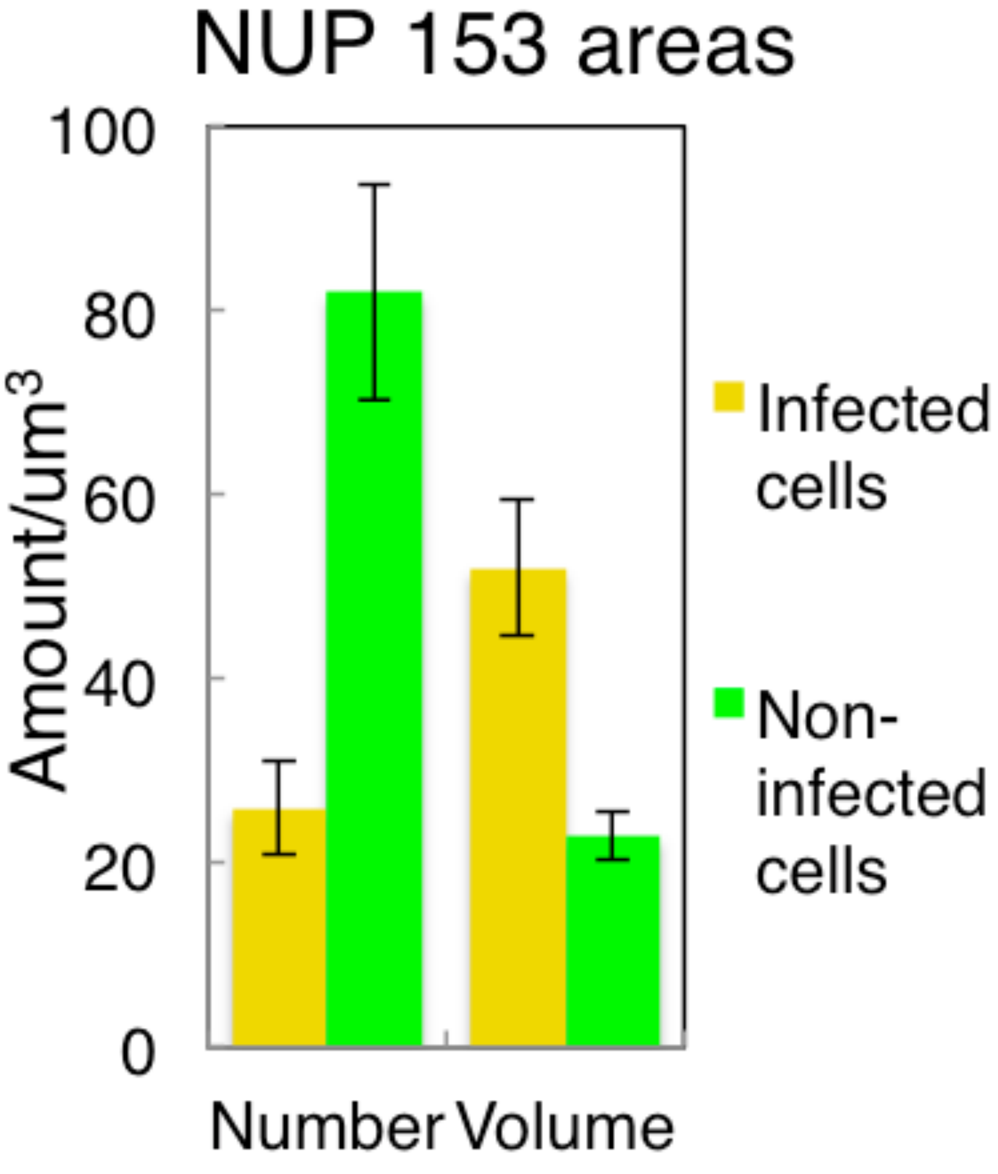

Figure S5. Chromatin distribution in non-infected cells. Transmission electron microscopy images of non-infected B cell nuclei. Inset shows enlarged view of the boxed areas containing the nuclear envelope and nuclear pore complexes. Scale bar, 2 µm.

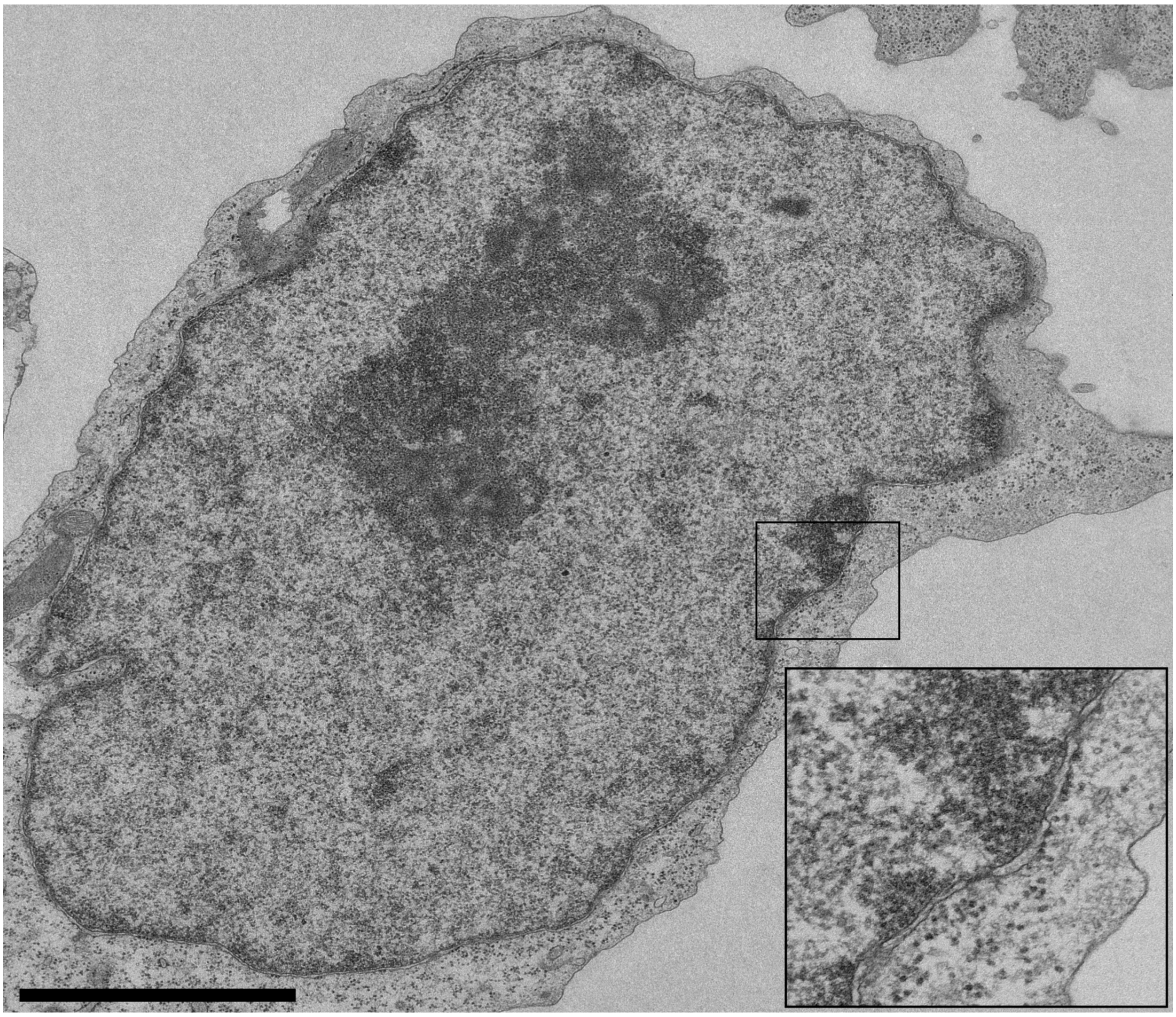

SUPPLEMENTAL VIDEOS

Video S1. Structure of marginalized chromatin. The 3D reconstruction of SXT images in Figs 1A and 1B, which show the distributions heterochromatin (blue) and nucleoplasm with VRC (yellow) in an infected cell, and those of heterochromatin (blue) and nucleoplasm without VRC (green) in a non-infected cell.

Video S2. 3D distribution of low-LAC gaps near to nuclear envelope. Animation of the computationally-generated skeletonized structures shown in Fig. 2 which demonstrate the existence of channels through the peripheral chromatin in infected (right) and non-infected (left) cells.

Video S3. 3D distribution of nucleocapsid proteins in low-density chromatin regions of nuclear periphery. Animation of the computationally-generated chromatin structure (grey) shown in Fig. 5B which demonstrate the presence of capsid proteins (magenta) in low-density chromatin channels through the peripheral chromatin.

SUPPLEMENTAL TEXT

Text S1. Materials and Methods

Infection of B cells by HSV-1.

Mouse B cells were infected with a herpes simplex virus type 1 (HSV-1, strain 17+), using a virus stock with a titer of 1 x 10^{10} plaque-forming units (pfu)/ml. These cells were infected at a density of 1 x 10^{6} /ml for 1 hr at a multiplicity of infection (MOI) of 0.5 or 5 plaque-forming units (pfu)/cell. There after the medium and the unbound virus were removed and the cells were washed with a sterile PBS. After infection the cells were maintained in 24-well plates in RPMI1640 (Gibco) supplemented with a 10 % fetal calf serum (PromoCell, Heidelberg, Germany), glutamine and gentamycin.

Flow cytometry, quantitative PCR and plaque assay.

For flow cytometry, the infected and uninfected cells were collected at 24 h post infection (p.i.), fixed with 2% PFA for 15 min and permeabilized with 0.1% Triton X-100 for 5 min. Permeabilized cells were stained with monoclonal antibody to the HSV protein ICP0 (Biofellows) and glycoprotein gD (Biofellows) at a dilution of 1:100, and with a monoclonal antibody to HSV glycoprotein gC (Ziegler et al., 1988) at a dilution of 1:50. For secondary antibody staining, Alexa Fluor 568 goat anti-mouse IgG (Invitrogen Molecular Probes) was used. Stained cells were collected with FACScan® flow cytometer (Becton Dickinson) and analyzed with a Cell Quest™ software.

For a quantitative analysis of the viral gene expression, nucleic acids were extracted from the B cells using a TRI Reagent (MRC Inc., Cincinnati, OH, USA) according to the manufacturer's instructions. The RNA from the DNAse-treated samples was reverse transcribed into cDNA using RevertAid H minus reverse transcriptase and random hexamer primers (Fermentas, currently Thermo Scientific). The quantitative real-time RT-PCR was accomplished using a Rotor-Gene Q real-time instrument and primers for the HSV genes ICP27 (UL54; alpha, immediate-early), ICP8 (UL29; beta, early) or VP16 (UL48; gamma, late) (A. Romanovskaya, H. Paavilainen, M. Nygårdas, H, Bamford, V. Hukkanen, and M. Poranen, PLoSOne **7**:e51019, 2012; H. Paavilainen, A. Romanovskaya, M. Nygårdas, D.H. Bamford, M. M. Poranen, and V. Hukkanen V, Innate Immun. **21**:349-357, 2015). The copy numbers were normalized to the GAPDH housekeeping gene copy numbers in the same sample (M. Nygårdas., H. Paavilainen, N. Müther, G.-H. Nagel, M. Röyttä, B. Sodeik, and H. Hukkanen, PLoS One **8**:e64200, 2013; Table S1).

Table S1. Quantitative PCR primers

HSV UL29 sense	5’-AAGCTGGTTGCGTTGGAG-3’
HSV UL29 antisense	5’-TTTCTGCTGAAGCAGTTCCA-3’
(Romanovskaya et al., 2012)	
HSV UL54 sense	5’-GTCCTGCGCTCCATCTCC-3’
HSV UL54 antisense	5’-GTCGTGCATGACCTGTGC-3’
HSV UL48 sense	5’-TTTGACCCGCGAGATCCTAT-3’
HSV UL48 antisense	5’-GCTCCGTTGACGAACATGAA-3’
(Paavilainen et al., 2014)	
GAPDH sense	5'-GCA AGG TCA TCC CAG AGC-3'
GAPDH antisense	5'-GGT CCT CAG TGT AGC CCA AG-3'
(Nygårdas et al., 2013)	

The shedding of infectious viruses from B cells was quantitated from the culture medium by plaque titration as described earlier (Romanovskaya et al., 2012). Briefly, the medium was collected at 4, 8, 12, 18, 24, 30, and 48 h p.i. and cultured tenfold diluted in Vero cells (ATCC; Manassas, VA, USA) in 12-well plates in DMEM supplemented with a 7 % fetal calf serum and 20 µg/ml human immunoglobulin G. analysis 5 "l of diluted DNA was deposited in triplicates.After three days in culture, the plaque-forming unit (pfu) values were counted from methanol-fixed wells, stained with 0.1 % crystal violet in ethanol.

Results

Infection of B cells by HSV-1

HSV-1 is a human pathogen with a broad host range in terms of both species and cell type (Spear and Longnecker, 2003; Eling, 2000). For SXT image acquisition, cells were placed in a (cylindrical) capillary tube of a diameter up to 15 μm. B cells were small enough to fit into these tubes.

In the nucleus, lytic infection by HSV-1 proceeds in a regulated cascade in which three temporal classes of viral genes are expressed: immediate-early, early and late (Honess and Roizman, 1974). The immediate-early gene products exert regulatory functions essential for the expression of both early and late genes, and immune-evasion functions (Allen and Everett, 1997; de Bruyn Kops et al., 1998; Quinlan et al., 1984; Watson et al., 1979). In order to characterize the HSV-1 replication in B cells, the infectivity of cells was analyzed by cytometry based on intracellular staining of immediate-early and late viral proteins (Fig. S1A). At 24 h post infection (p.i.), this analysis indicated that viral glycoproteins C (gC) and D (gD) were detectable in 1.0 ± 0.6 % and 2.2 ± 0.5 % of the cells, respectively, infected at MOI 0.5 pfu/cell, and in 4.5 ± 2.1 %, and 10.6 ± 1.0 % of the cells when infected at MOI 5. Moreover, the percentages of ICP0-expressing cells were 1.6 ± 0.5% at MOI 0.5, and 7.2 ± 1.6% at MOI 5. These data indicated that ICP0 and viral glycoproteins were expressed in infected B cells at 24 h p.i. in an MOI-dependent manner.

We next determined the progress of infection in B cells by quantitating the expression of the immediate-early (ICP27; UL54), early (ICP8; UL29) and late (alpha trans-inducing factor, aTIF; VP16; UL48) RNAs by real-time RT-PCR (qPCR) (Fig. S1B). The glyceraldehyde-3-phosphate dehydrogenase (GAPDH)-normalized relative copy numbers of VP16 (UL48) mRNA increased 12.8-fold from hours 4 to 24 p.i. in cells infected at MOI 0.5. For infection

at MOI 5, the relative UL48 mRNA values increased 27-fold between hours 4 and 24 p.i. The relative copy numbers of ICP27 (UL54) mRNA increased 2.3-fold between 4 and 24 h p.i. in cells infected at MOI 0.5, and when using the MOI=5, the UL54 mRNA values increased 5.3-fold. The relative copy numbers of the essential ICP8 (UL29) mRNA increased 2.3-fold (MOI 0.5) and 5.5-fold (MOI 5) between 4 h and 24 h p.i. These data indicate that all three viral mRNAs could be detected in cells at 4 h p.i., and, subsequently, by 24 h p.i., levels of mRNAs increased in an MOI- and time-dependent manner. Notice that the presence and increase of VP16 mRNA indicated that late genes, the last set to be transcribed, were indeed expressed in infected B cells.

In order to follow the time course of infection in B cells, we quantified the virus yield by the plaque assay from culture supernatants collected at 4, 8, 12, 18, 24, 30, and 48 h p.i. at MOI 5. The increased presence of progeny viruses in supernatant was detected at 12 h p.i and the amount of viruses increased time-dependently until 24 h p.i. The amount of virus shedding decreased at later times (Fig. S2).

In conclusion, we detected the presence of viral proteins, as well as the expression of viral lytic genes of all three phases of the replication cycle, and substantial production of the progeny virus from the B cells. This suggests that HSV-1 not only could enter and infect B cells but that its replication cycle was completed. Based on these findings, we decided to use an MOI of 5 and the time point 24h in all our subsequent experiments of this study.
